# Genetic architecture of the limbic white matter microstructure in aging and Alzheimer's Disease

**DOI:** 10.1002/alz.71630

**Published:** 2026-07-06

**Authors:** Anna S Lorenz, Aditi Sathe, Yisu Yang, Alaina Durant, Yiyang Wu, Michael E. Kim, Chenyu Gao, Nancy R. Newlin, Karthik Ramadass, Praitayini Kanakaraj, Nazirah Mohd Khairi, Zhiyuan Li, Tianyuan Yao, Yuankai Huo, Logan Dumitrescu, Niranjana Shashikumar, Kimberly R. Pechman, Shannon L. Risacher, Lori L. Beason‐Held, Yang An, Konstantinos Arfanakis, Guray Erus, Christos Davatzikos, Mohamad Habes, Di Wang, Duygu Tosun, Arthur W. Toga, Paul M. Thompson, Elizabeth C. Mormino, Panpan Zhang, Kurt Schilling, Marilyn Albert, Walter Kukull, Sarah A. Biber, Bennett A. Landman, Sterling C. Johnson, Barbara Bendlin, Julie Schneider, David A. Bennett, Angela L. Jefferson, Susan M. Resnick, Andrew J. Saykin, Jennifer E. Below, Timothy J. Hohman, Derek B. Archer, Alzheimer's Disease Neuroimaging Initiative (ADNI), The BIOCARD Study Team (BIOCARD), The Alzheimer's Disease Sequencing Project (ADSP)

**Affiliations:** ^1^ Vanderbilt Memory and Alzheimer's Center Vanderbilt University School of Medicine Nashville Tennessee USA; ^2^ Vanderbilt Genetics Institute Vanderbilt University Medical Center Nashville Tennessee USA; ^3^ Department of Computer Science Vanderbilt University Nashville Tennessee USA; ^4^ Department of Electrical and Computer Engineering Vanderbilt University Nashville Tennessee USA; ^5^ Department of Neurology Vanderbilt University Medical Center Nashville Tennessee USA; ^6^ Vanderbilt Brain Institute Vanderbilt University Medical Center Nashville Tennessee USA; ^7^ Department of Radiology and Imaging Sciences Indiana University School of Medicine Indianapolis Indiana USA; ^8^ Indiana Alzheimer's Disease Research Center Indiana University School of Medicine Indianapolis Indiana USA; ^9^ Laboratory for Behavioral Neuroscience National Institute on Aging National Institutes of Health Baltimore Maryland USA; ^10^ Department of Biomedical Engineering Illinois Institute of Technology Chicago Illinois USA; ^11^ Rush Alzheimer's Disease Center Rush University Medical Center Chicago Illinois USA; ^12^ Department of Diagnostic Radiology Rush University Medical Center Chicago Illinois USA; ^13^ Department of Radiology University of Pennsylvania Philadelphia Pennsylvania USA; ^14^ Neuroimage Analytics Laboratory and Biggs Institute Neuroimaging Core Glenn Biggs Institute for Alzheimer's and Neurodegenerative Diseases University of Texas Health Science Center San Antonio Texas USA; ^15^ Department of Radiology and Biomedical Imaging University of California San Francisco California USA; ^16^ Laboratory of Neuroimaging USC Stevens Institute of Neuroimaging and Informatics Keck School of Medicine University of Southern California Los Angeles California USA; ^17^ Imaging Genetics Center Mark and Mary Stevens Institute for Neuroimaging and Informatics Keck School of Medicine University of Southern California Marina del Rey California USA; ^18^ Department of Neurology and Neurological Sciences Stanford University School of Medicine Stanford California USA; ^19^ Department of Biostatistics Vanderbilt University Medical Center Nashville Tennessee USA; ^20^ Department of Radiology & Radiological Sciences Vanderbilt University Medical Center Nashville Tennessee USA; ^21^ Vanderbilt University Medical Center Vanderbilt University Institute of Imaging Science Nashville Tennessee USA; ^22^ Department of Neurology Johns Hopkins School of Medicine Baltimore Maryland USA; ^23^ National Alzheimer's Coordinating Center University of Washington Seattle Washington USA; ^24^ Department of Biomedical Engineering Vanderbilt University Nashville Tennessee USA; ^25^ Wisconsin Alzheimer's Disease Research Center School of Medicine and Public Health University of Wisconsin Madison Wisconsin USA; ^26^ Wisconsin Alzheimer's Institute School of Medicine and Public Health University of Wisconsin Madison Wisconsin USA; ^27^ Department of Medicine Vanderbilt University Medical Center Nashville Tennessee USA

**Keywords:** Alzheimer's disease, aging, diffusion MRI, GWAS, harmonization, white matter microstructure

## Abstract

**INTRODUCTION:**

Limbic white matter (WM) abnormalities are prevalent in aging and Alzheimer's disease (AD), but genetic drivers are unclear.

**METHODS:**

In 2614 older adults (mean age ± SD: 73.7 ± 9.8 years; 26% cognitively impaired) from seven harmonized cohorts enriched for cognitive impairment, we quantified free‐water–corrected diffusion MRI (dMRI) metrics in seven limbic tracts. We estimated single nucleotide polymorphism (SNP) heritability, performed cohort genome‐wide association studies (GWASs) with meta‐analysis, evaluated shared genetic architecture and enriched pathways, and assessed AD relevance using brain RNA‐seq data.

**RESULTS:**

Limbic WM is heritable (*h^2^* = 0.26–0.60; *p_FDR_* < 0.05). Meta‐GWAS identified six loci (*p* < 5 × 10^−^^8^), including a signal implicating *CDH19*, an oligodendrocyte‐enriched cell‐adhesion gene. Additional loci were near the *KC6*, *SENP5*, *RORA*, *FAM107B*, and *MIR548A1* genes. In brain tissue, *RORA*, *FAM107B*, and *KC6* expression was associated with cognition and AD neuropathology. Results converged on insulin and immune biology and shared genetic architecture with lipid and cardiovascular traits.

**DISCUSSION:**

Limbic WM microstructure is genetically influenced and links oligodendrocyte and vascular‐inflammatory biology to AD‐relevant outcomes.

## Highlights

- Limbic free‐water (FW)–corrected white matter (WM) integrity is heritable in late‐life aging
- Six genome‐wide loci discovered for limbic WM traits in Alzheimer's disease (AD)–enriched cohorts
- Chr18 locus implicates *CDH19*, linking oligodendrocyte biology to WM microstructure
- *RORA*, *FAM107B*, and *KC6* expression tracks cognitive decline and AD neuropathology

## 1 BACKGROUND

The integrity of nerve fibers and the myelination of axons, which characterize white matter (WM) microstructure, are essential for cognitive processing by enabling communication across brain regions.^1^, ^2^ However, WM undergoes substantial changes during aging and in neurodegenerative diseases such as Alzheimer's disease (AD).^3^ Although AD has been viewed as a disorder primarily affecting gray matter, emerging evidence highlights WM abnormalities, including axonal loss and demyelination.^4^, ^5^, ^6^ These changes, particularly within the limbic system, are prevalent across the AD diagnostic continuum,^7^ with accelerated degeneration in individuals exhibiting abnormal aging^8^ and carriers of the apolipoprotein E (*APOE*) ε4 allele.^9^ They can manifest before the appearance of amyloid beta (Aβ) plaques and neurofibrillary tangles,^5^ are detectable up to 22 years before symptom onset,^10^, ^11^ and contribute to cognitive decline independently of gray matter atrophy.^12^

By estimating the directionality of water diffusion in the brain, diffusion magnetic resonance imaging (dMRI) enables in vivo quantification of WM microstructure through metrics including fractional anisotropy (FA_CONV_), axial diffusivity (AxD_CONV_), mean diffusivity (MD_CONV_), and radial diffusivity (RD_CONV_).^13^, ^14^, ^15^, ^16^ FA_CONV_ reflects the directional dependence of diffusion, with higher values typically indicating compact tracts. AxD_CONV_ measures diffusion along the primary axis of fibers, where lower values can indicate axonal damage. MD_CONV_ represents the average diffusion rate across tissue and extracellular compartments and is sensitive, but not specific, to multiple microstructural properties. RD_CONV_ measures diffusion perpendicular to the primary axis, with higher values often suggesting demyelination.^13^, ^14^, ^15^, ^16^ Conventional metrics derived from single‐tensor models can be confounded by partial volume effects of extracellular free water (FW), such as cerebrospinal fluid. Advanced bi‐tensor models address this by correcting these metrics for FW contributions, allowing differentiation between extracellular (i.e., FW) and intracellular contributions (FA_FWcorr_, AxD_FWcorr_, RD_FWcorr,_ MD_FWcorr_) to diffusion.^17^ FW‐corrected measures provide tissue‐compartment analogs of conventional metrics, yielding more biologically specific indices of WM microstructure, and better capture WM neurodegenerative patterns in AD.^7^, ^8^, ^9^, ^12^ However, they should not be interpreted as direct markers of any single histopathologic process.

Measurements of WM microstructure in vivo have enabled the examination of underlying biological mechanisms and demonstrated that WM is influenced by genetics. Twin studies reported high heritability for dMRI metrics (up to 82%), indicating substantial genetic contributions to variations in WM.^18^, ^19^ Genome‐wide association studies (GWASs) in cognitively unimpaired populations, such as the UK Biobank, have identified more than 100 genomic regions influencing WM microstructure.^20^, ^21^ However, these studies may not fully capture genetic pathways specific to neurodegeneration due to the underrepresentation of cognitively impaired individuals and those at risk for AD. Investigations focused on AD have implicated candidate genes, such as *APOE* and *BIN1*, in WM alterations.^22^, ^23^, ^24^, ^25^ In addition, a genetic predisposition for AD and several AD‐risk variants in *TMEM106B*, *PTK2B*, *WNT3*, and *APOE* are associated with microstructure.^26^, ^27^

Nevertheless, a more comprehensive understanding of the genetic architecture influencing WM microstructural differences in older adults, particularly those at risk for or with AD, remains limited due to the scarcity of combined diffusion and genetic data. Therefore, examining genetic influences on WM microstructure in cohorts enriched for cognitive impairment is critical for advancing our understanding of neurodegenerative processes in aging and in populations at increased risk for AD. Large‐scale harmonization efforts, such as the Alzheimer's Disease Sequencing Project Phenotype Harmonization Consortium (ADSP‐PHC), in combination with advanced FW‐corrected dMRI methods, present an unprecedented opportunity to investigate these imaging genetic associations.

This study assessed the role of genetic factors in WM microstructure across seven tracts of the limbic system, including the cingulum, fornix, inferior longitudinal fasciculus (ILF), uncinate fasciculus (UF), and the transcallosal tracts of the inferior temporal gyrus (ITG), middle temporal gyrus (MTG), and superior temporal gyrus (STG). These tracts are integral to memory function and substantially alter in aging, cognitive impairment, and AD‐related neurodegeneration.^7^, ^12^, ^24^ By analyzing harmonized FW‐corrected data from 2614 older adults (26% cognitively impaired) across seven aging cohorts, we aim to identify novel biological pathways associated with limbic WM microstructure, providing insights into the mechanisms of WM degeneration in the context of aging, cognitive impairment, and AD risk.

**RESEARCH IN CONTEXT**

1. **Systematic review**: We surveyed prior diffusion magnetic resonance imaging (MRI) studies of limbic white matter (WM) degeneration in aging and Alzheimer's disease (AD), including evidence that free‐water (FW)–corrected metrics improve biological specificity over conventional tensor measures. We reviewed large‐scale genome‐wide association studies (GWASs) of WM microstructure (primarily in population‐based, cognitively unimpaired cohorts) and studies linking AD risk genetics, vascular‐metabolic factors, and immune dysregulation to WM injury and cognitive decline.
2. **Interpretation**: Using harmonized multi‐cohort data enriched for cognitive impairment, we show that FW‐corrected limbic WM is substantially heritable and identified six genome‐wide loci underlying tract‐specific microstructure. A strong chromosome 18 signal implicates *CDH19*, consistent with oligodendrocyte and myelin biology. Integration with human brain transcriptomics demonstrated that *RORA*, *FAM107B*, and *KC6* expression relate to cognitive decline and AD neuropathology, strengthening relevance to AD mechanisms. Post‐GWAS results converge on insulin signaling, immune processes, and neurotrophic pathways, and reveal shared genetics with lipid and cardiovascular traits.
3. **Future directions**: Further directions include replication in multi‐ancestry samples, incorporating longitudinal WM microstructural changes and validation of cell‐type mechanisms in oligodendrocytes and neuroinflammation.

## 2 METHODS

### 2.1 Participants

dMRI and genetic data were leveraged from seven cohorts, including the Alzheimer's Disease Neuroimaging Initiative (ADNI),* Biomarkers of Cognitive Decline Among Normal Individuals (BIOCARD), Baltimore Longitudinal Study of Aging (BLSA), National Alzheimer's Coordinating Center (NACC), Religious Orders Study and Rush Memory and Aging Project (ROSMAP), Vanderbilt Memory and Aging Project (VMAP), and the Wisconsin Registry for Alzheimer's Prevention (WRAP).

Data used in the preparation of this article were obtained from the ADNI database (adni.loni.usc.edu). The ADNI was launched in 2003, as a public–private partnership, led by Principal Investigator Michael W. Weiner, MD. The primary goal of ADNI has been to test whether serial MRI, positron emission tomography (PET), other biological markers, and clinical and neuropsychological assessment can be combined to measure the progression of mild cognitive impairment (MCI) and early AD.^28^ ADNI‐GO, ADNI2, and ADNI3 phases with dMRI data were included in this study. BIOCARD began data collection in 1995, at the National Institute of Mental Health (NIMH) and was later transferred to Johns Hopkins University with the goal of identifying preclinical biomarkers of cognitive decline and investigating variables that can predict future progression to AD among cognitively normal middle‐aged individuals. Participants undergo extensive longitudinal evaluations, including neuropsychological testing, MRI scans, and collection of blood and cerebrospinal fluid samples.^29^ The BLSA neuroimaging substudy began data collection in 1994, and it included dementia‐free participants 55 to 85 years of age, with up to 10 years of prospective data collection at baseline. In 2009, the cohort was expanded to include BLSA participants 20 to 85 years of age, as well as 3T MRI‐based acquisition of dMRI data. The NACC maintains a centralized data repository for the National Institute on Aging's (NIA) Alzheimer's Disease Research Centers (ADRC) Program, which currently includes 33 centers and 4 exploratory centers across the United States.^30^, ^31^, ^32^ The ROS cohort, which began in 1994, is an ongoing longitudinal study collecting clinical‐pathological data on aging and AD. Participants in ROS are 65+ years of age Catholic nuns, priests, and brothers from various groups throughout the United States.^33^ MAP is a longitudinal study that started in 1997, recruiting cognitively unimpaired participants.^33^ The VMAP cohort began longitudinal data collection in 2012, with the goal of understanding the relationship between vascular health and brain aging in older adults with MCI.^34^ The WRAP cohort began data collection in 2001, focusing on middle‐aged adults with a parental history of AD. In 2004, the study expanded to include a control group of individuals without a parental history of AD. The primary objective of WRAP is to identify early biomarkers and risk factors for AD before clinical symptoms emerge.^35^, ^36^

For all cohorts, participants provided written informed consent, and research was conducted in accordance with approved institutional review board protocols. Secondary analysis of these data was approved by the Vanderbilt University Medical School Institutional Review Board. The study included 2614 participants who were self‐reported non‐Hispanic White individuals between 50.1 and 100.9 years of age (mean age ± SD: 73.7 ± 9.8 years), and 57% were female. Table 1 provides an overview of data included for this study from the ADNI, BIOCARD, BLSA, NACC, ROSMAP, VMAP, and WRAP cohorts.

**TABLE 1:** Participant characteristics by cohort.

	ADNI	BIOCARD	BLSA	NACC	ROSMAP	VMAP	WRAP	Total
**Number of participants**	491	104	399	659	496	247	218	2614
**Number of female participants** (%)	236 (48%)	66 (63%)	210 (53%)	371 (56%)	381 (77%)	92 (37%)	143 (66%)	1499 (57%)
**Age**, years	74.7 (7.6)	73.4 (6.8)	73.2 (9.7)	71.3 (10.5)	81.1 (7.2)	73.7 (7.1)	62.4 (6.1)	73.7 (9.8)
**Education**, years	16.4 (2.6)	17.5 (2.2)	17.1 (2.4)	15.6 (3.0)	15.8 (3.2)	15.9 (2.7)	16.8 (2.8)	16.2 (2.9)
**Number of *APOE* ε4 positive** (%)	184 (37%)	34 (33%)	91 (23%)	281 (43%)	104 (21%)	88 (36%)	68 (31%)	850 (33%)
**Number of baseline diagnosis**								
CU (%) / MCI (%) / AD (%)	282 (57%) / 171 (35%) / 38 (8%)	82 (79%) / 20 (19%) / 2 (2%)	395 (99%) / 3 (0.8%) / 1 (0.2%)	416 (63%) / 152 (23%) / 91 (14%)	403 (81%) / 88 (18%) / 5 (1%)	147 (60%)/ 99 (40%)/ 1 (0.4%)	214 (98%)/ 3 (1.4%)/ 1 (0.5%)	1939 (74%)/ 536 (21%)/ 139 (5%)

*Note*: Values denoted as mean (SD) or frequency (percentage).

Abbreviations: AD, Alzheimer's disease; ADNI, Alzheimer's Disease Neuroimaging Initiative; *APOE*, apolipoprotein E; BIOCARD, Predictors of Cognitive Decline Among Normal Individuals; BLSA, Baltimore Longitudinal Study of Aging; CU, cognitively unimpaired; MAP, Memory and Aging Project; MARS, Minority Aging Research Study; MCI, mild cognitive impairment; NACC, National Alzheimer's Coordinating Center; ROS, Religious Orders Study; VMAP, Vanderbilt Memory and Aging Project; WRAP, Wisconsin Registry for Alzheimer's Prevention.

### 2.2 dMRI acquisition and preprocessing

Across all cohorts, we had 78 different dMRI acquisition protocols; Table S1 provides relevant parameters (e.g., number of directions, *b*‐values, resolution). dMRI data from all cohorts were preprocessed using the *PreQual* pipeline, which addresses motion, distortions, and eddy currents and performs slice‐wise imputation.^37^, ^38^ Imaging sessions were inspected manually by evaluating reports outputted by the *PreQual* pipeline. DTIFIT was used for the remaining data to compute conventional dMRI metrics, including FA_CONV_, AxD_CONV_, MD_CONV_, and RD_CONV_. To account for FW content in each voxel, we calculated FW‐corrected metrics, including FA_FWcorr_, AxD_FWcorr_, MD_FWcorr_, RD_FWcorr_, and FW.^17^ Symmetric normalization and linear interpolation were conducted using the Advanced Normalization Tools (ANTs) package to obtain a standard space representation of these maps by non‐linearly registering the FA_CONV_ map to FMRIB58_FA atlas.^39^ The obtained warp from the registration was applied to all other microstructural maps. Individuals with significant age‐regressed outliers (± 5 SD) in WM tract microstructural values were excluded.^40^

### 2.3 WM tractography templates

Tractography templates for this study were leveraged from existing resources^12^, ^41^, ^42^ and can be retrieved from a publicly available Zenodo repository.^43^ The focus for this study was on seven WM tracts within the limbic system that are relevant to memory and are known to be vulnerable in aging and neurodegeneration, including AD, specifically the cingulum, fornix, ILF, UF, as well as ITG, MTG, and STG transcallosal tracts.^44^

### 2.4 Data harmonization

For the dMRI data, a region of interest (ROI) approach was employed to calculate mean conventional and FW‐corrected dMRI metrics for all tractography templates for each participant. These values were then harmonized using the *Longitudinal ComBat* package in R (version 4.1.0)^45^ applied to our entire in‐house longitudinal dataset, including 5144 participants across 10,346 imaging timepoints. This harmonization process utilized a batch variable that had varying levels of specificity depending on the cohort. Parameters used to create the batch variable can be found in Table S2.^7^, ^8^, ^9^ The batch variable accounted for various parameters across our cohorts, including scanner name, magnet strength, number of *b*‐values/b‐vectors, and resolution; this was optimized to reduce the number of batches while simultaneously accounting for parameters we most anticipated to explain between‐batch heterogeneity. In total, we accounted for 34 unique batching levels. Additional covariates for the harmonization included mean‐centered age, mean‐centered age squared, education, race/ethnicity, cognitive status (cognitively unimpaired or cognitively impaired), *APOE* ε4 positivity, *APOE* ε2 positivity, and the interaction of mean‐centered age and cognitive status.

The harmonized values were then scaled by their SD. Next, the dataset was filtered to include only participants with genetic data and further narrowed to the baseline time point for cross‐sectional analysis. We ultimately used FW‐corrected dMRI measures across seven limbic WM tracts (35 dMRI measures) for each participant.

### 2.5 Genetic data quality control and imputation

Genetic data were collected with various genotyping arrays across and within cohorts (ADNI: Illumina Human610‐Quad BeadChip, Illumina HumanOmniExpress BeadChip, Illumina Omni 2.5 M, Illumnia Global Screening Array v2; BIOCARD: Illumina OmniExpress; BLSA: Illumina HumanOmni2.5 BeadChip, Illumina HumanOmniExpress BeadChip; NACC: several different arrays were used to collect genetic data—acquisition of all genetic data is outlined on the NACC website [https://naccdata.org/nacc‐collaborations/partnerships]; ROSMAP: Global Screening Array‐24 v3.0 BeadChip, Affymetrix GeneChip 6.0, Illumina HumanOmniExpress; VMAP: Illumina HumanOmniExpress; WRAP: Illumina Human610, Illumina OmniExpress).All genetic raw data underwent the same robust quality control and imputation pipelines.^46^ Variants that had a genotyping rate less than 95%, a minor allele frequency (MAF) less than 1%, or deviated from Hardy‐Weinberg Equilibrium (*p* < 1×10^−6^) were removed. In addition, participants were excluded if genotyping efficiency was poor (missing > 1% of variants), if cryptic relatedness was present (PIHAT > 0.25), or if the reported and genotypic sex were not concordant. Imputation was performed on the University of Michigan Imputation Server using the TOPMed reference panel (hg38) with SHAPEIT phasing.^47^ Data were filtered to exclude variants with low imputation quality (R^2^ < 0.80), duplicated/multi‐allelic variants, and MAF < 1%. Principal components analysis was conducted, and genetic ancestry outliers were excluded. Eight pairs of individuals were related across cohorts and were subsequently removed from the respective cohort with more individuals, namely BLSA, NACC, and ADNI (PIHAT > 0.25).

### 2.6 Statistical analyses

#### 2.6.1 SNP‐heritability tests

Single nucleotide polymorphism (SNP) heritability was estimated for each FW‐corrected dMRI metric using genome‐wide complex trait analysis (GCTA). This method uses restricted maximum likelihood and genetic relatedness matrices to calculate heritability estimates.^48^ To account for multiple comparisons, results were adjusted across phenotypes using the false discovery rate (FDR) procedure.^49^

#### 2.6.2 GWAS testing and meta‐analysis

GWAS in self‐reported non‐Hispanic White participants were conducted separately for each cohort (*N* = 7) for each dMRI metric (*N* = 5) of each of the limbic WM tracts (*N* = 7) using PLINK software (Version 1.9, https://www.cog‐genomics.org/plink/1.9). All models included covariates for age, sex, and the first three genetic ancestry principal components. Next, we performed a meta‐analysis across cohorts for each dMRI metric using genome‐wide association meta‐analysis (GWAMA).^50^ The significance threshold was set a priori to *p* < 5 × 10^−8^. We evaluated suggestive loci that had a *p* < 1 × 10^−5^. Reported genome‐wide associations were filtered to include those present in at least six of seven cohorts. Variants were mapped to genes by proximity using the Open Targets platform.

#### 2.6.3 eQTL analyses, replication, and databases

Expression quantitative trait locus (eQTL) analyses were conducted on variants reaching genome‐wide significance using the Genotype‐Tissue Expression (GTEx) portal (https://gtexportal.org). Because expression regulation is broadly correlated across tissues and sample sizes for brain tissues in GTEx are small and therefore underpowered to detect modest effects, we considered all GTEx tissues in our post‐GWAS eQTL analyses.

To verify whether the identified variants have previously been associated with brain traits, genome‐wide significant variants were validated using the Oxford Brain Imaging Genetics Server (BIG40) (https://open.win.ox.ac.uk/ukbiobank/big40/)^51^ and ENIGMA‐VIS (https://enigma‐brain.org/).^52^

The identified variants and their mapped genes were further explored using several databases, including GeneCards (https://www.genecards.org), Agora (https://agora.adknowledgeportal.org), and Open Targets (https://www.opentargets.org). A literature search was also conducted using PubMed and Web of Science to provide further biological context.

#### 2.6.4 Association analysis with cognitive outcomes and AD pathologies using ROSMAP bulk RNA sequencing data

Processed bulk RNAseq data in the ROSMAP cohort from three brain tissues (Table S3), including the dorsolateral prefrontal cortex (DLPFC), the posterior cingulate cortex (PCC), and the head of the caudate nucleus (CN),^53^ were used to fit linear regression models (for cross‐sectional outcomes) and linear mixed‐effects models (for longitudinal outcomes) to test associations between cognitive function and AD pathologies with expression of the genes closest to the identified genome‐wide significant variants in the meta‐analyzed GWAS.

Overall cognitive function was represented using a global cognitive score, which was derived by converting raw scores from 19 cognitive tests to z‐scores and subsequently averaged. For cross‐sectional cognitive function, the global cognitive score at the last visit before death was used. For the longitudinal model, cognitive trajectory was quantified in a mixed‐effects regression model. Cognitive trajectory was estimated as the individual‐specific annual rate of change in global cognition. AD pathologies included Aβ load (immunohistochemistry staining), neurofibrillary tangles (silver staining), tau tangle (immunohistochemistry staining), and neuritic plaque pathology (silver staining) (https://www.radc.rush.edu/docs/var/overview.htm?category=Pathology).

For all cross‐sectional outcomes, covariates included age at death, sex, postmortem interval (PMI), and interval between last visit and death. For the longitudinal models, in addition to the same set of covariates, time was modeled as the number of years between each visit and the final visit. To account for multiple comparisons, results were adjusted using the FDR correction.^49^

#### 2.6.5 Gene‐ and pathway‐level analysis

The Multimarker Analysis of GenoMic Annotation (MAGMA v1.09) software was used to conduct gene‐ and pathway‐level analyses.^54^ Results were adjusted using FDR correction.^49^

#### 2.6.6 Genetic covariance analysis and local genetic covariance analysis

The meta‐analysis results for all dMRI metrics were used to perform genetic covariance analyses with the GWAS summary statistics of 65 complex traits (Table S4) as well as 3143 brain traits^55^ derived from the UK Biobank using the Genetic Covariance Analyzer (GNOVA) software.^56^ To further investigate local genetic covariance for significant associations, we used the Super Genetic Covariance Analyzer (SUPERGNOVA) software.^57^ Results were adjusted across dMRI metrics and traits using the FDR procedure.^49^

#### 2.6.7 Transcriptome‐wide association testing

By leveraging predictive models that aggregate evidence across SNPs, transcriptome‐wide association studies (TWASs) increase the power to detect associations with WM microstructure that might have been missed with the GWAS approach. S‐PrediXcan was used to examine associations between genetically predicted gene expression and dMRI metrics, leveraging precomputed predictive gene expression models from all GTEx tissues.^58^ To account for multiple comparisons, results were adjusted across genes and tissues using the FDR procedure.^49^

## 3 RESULTS

### 3.1 Heritability of limbic WM microstructure

**FIGURE 1:**
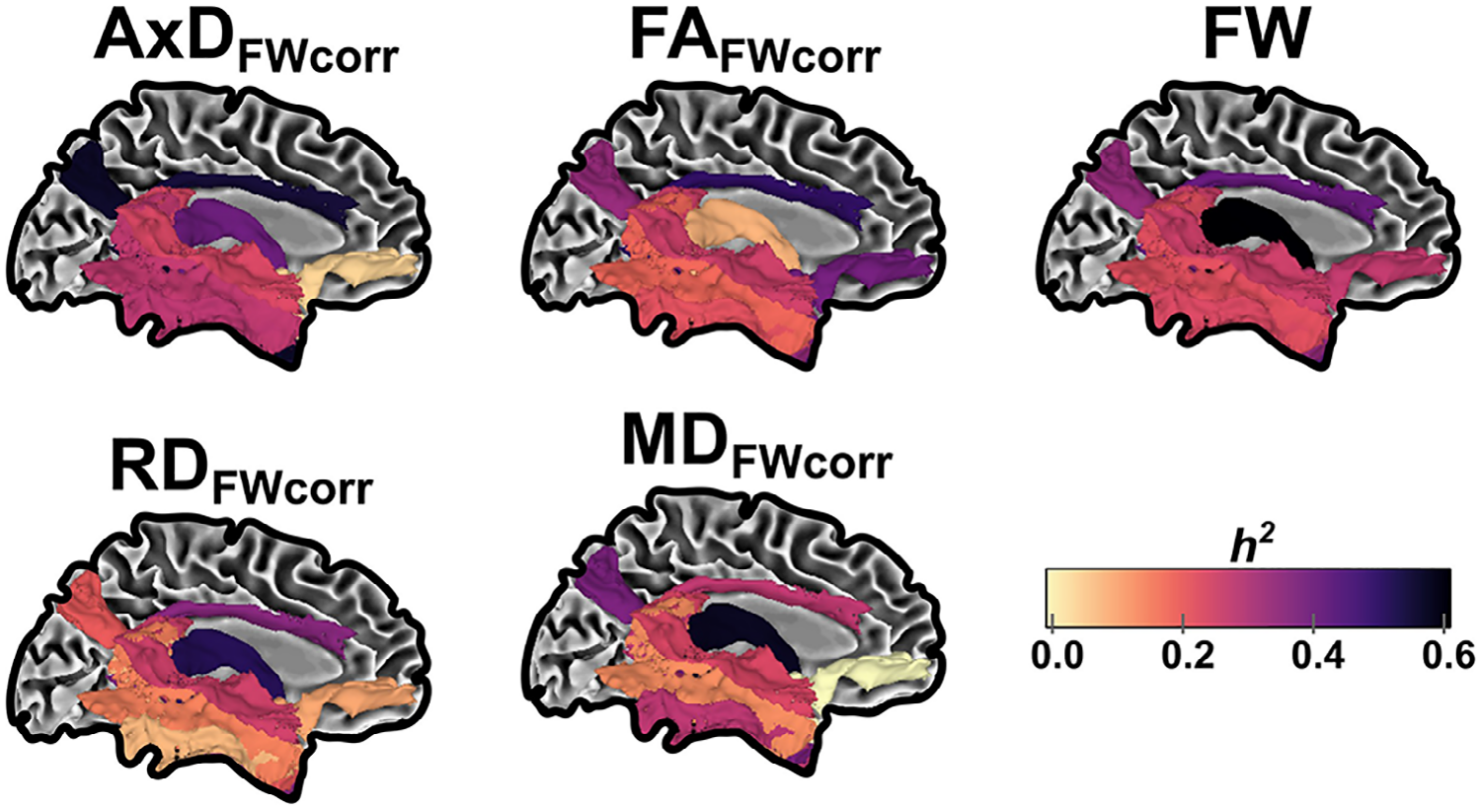
SNP heritability estimates for limbic white matter (WM) microstructure. SNP heritability of 35 FW‐corrected dMRI metrics from seven WM tracts in the limbic system. Abbreviations: AxD, axial diffusivity; dMRI, diffusion magnetic resonance imaging; FA, fractional anisotropy; FW_corr_, free‐water corrected; MD, mean diffusivity; RD, radial diffusivity; WM, white matter.

Fifteen of 35 FW‐corrected dMRI metrics across all limbic tracts were heritable with SNP‐heritability estimates ranging from 0.26 to 0.60 (*p_FDR_* < 0.05, Figure 1, Table S5). Specifically, all dMRI metrics of the cingulum, as well as four of five metrics of the fornix and the ILF showed significant heritability.

### 3.2 Genome‐wide significant signals in proximity to the genes *CDH19*, *KC6*, *SENP5*, *RORA*, *FAM107B*, and *MIR548A1* were associated with limbic WM microstructure

**FIGURE 2:**
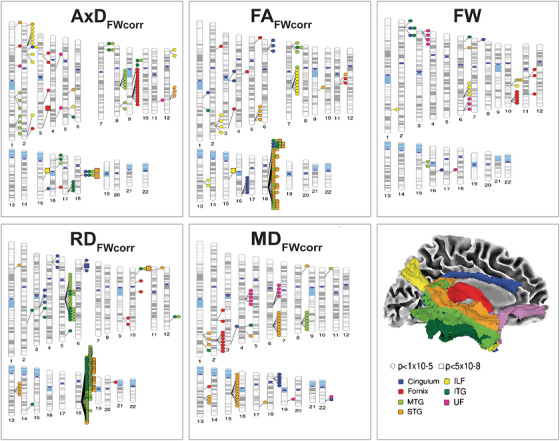
Ideogram of genomic signals associated with white matter (WM) microstructure. Ideogram of selected genomic regions associated with dMRI metrics. Shape codes for the *p*‐value threshold (*p* < 5 × 10^−8^; *p* < 1 × 10^−5^), color codes for the WM tract. The 500 associations between genetic variants and dMRI metrics with the smallest *p*‐value are represented in this plot. This figure was created using PhenoGram from Richie Lab Visualizations (https://visualization.ritchielab.org/phenograms/plot). Abbreviations: AxD, axial diffusivity; dMRI, diffusion‐weigthed magnetic resonance imaging; FA, fractional anisotropy; FW, free water; ILF, inferior longitudinal fasciculus; ITG, inferior temporal gyrus transcallosal tract; MD, mean diffusivity; MTG, middle temporal gyrus transcallosal tract; RD, radial diffusivity; STG, superior temporal gyrus transcallosal tract; UF, uncinate fasciculus; WM, white matter.

**FIGURE 3:**
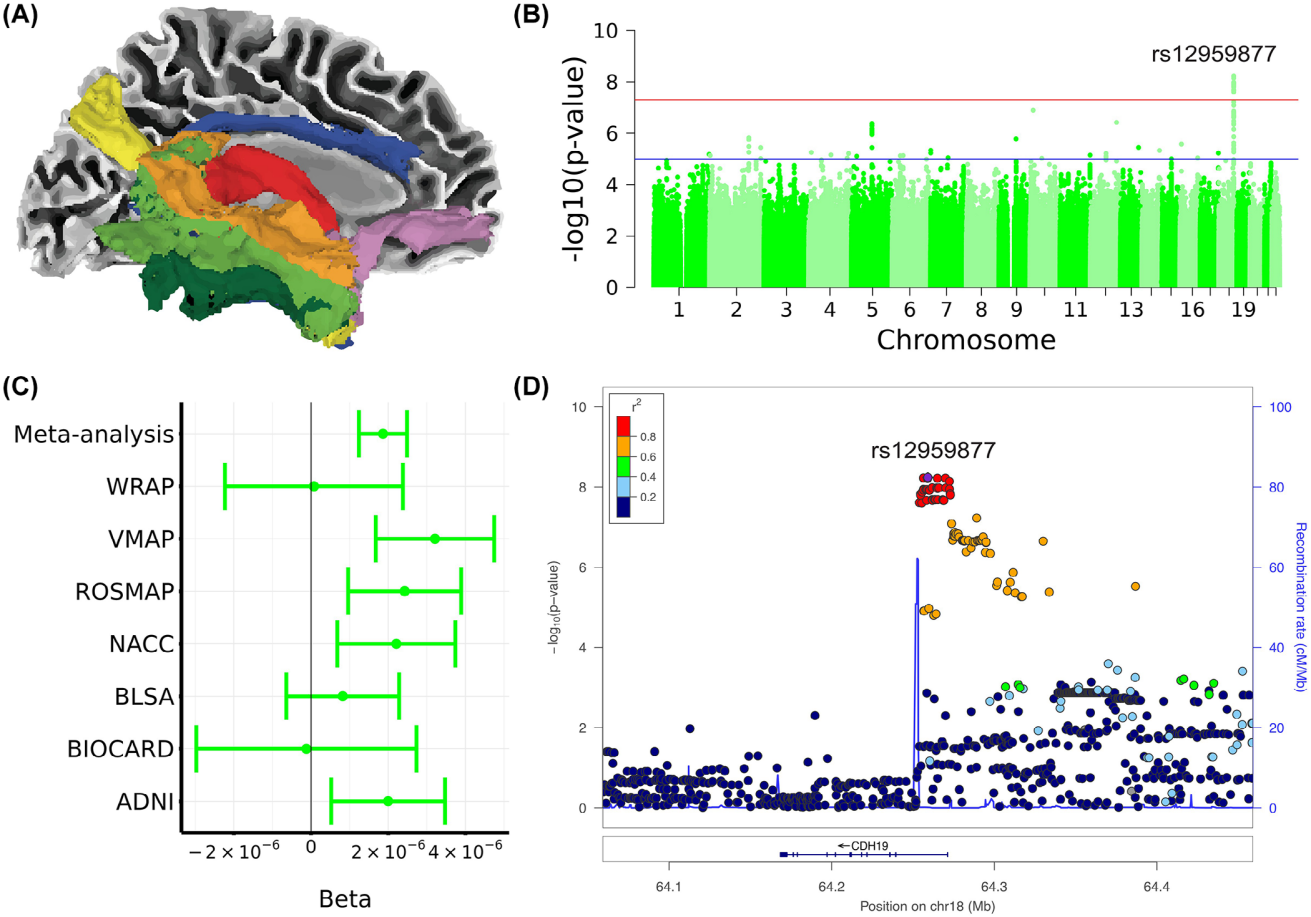
Genome‐wide significant locus near CDH19 associated with medial temporal gyrus (MTG) free‐water corrected radial diffusivity (RD_FWcorr_). (A) Location of the seven limbic white matter (WM) in the brain, with the MTG colored in light green. (B) Manhattan plot of GWAS results for MTG RD_FWcorr_. The red line indicates genome‐wide significance (*p* < 5 × 10^−8^). The blue line indicates suggestive significance (*p* < 1 × 10^−5^). (C) Forest plot for the effect of variant rs12959877 for each cohort. (D) LocusZoom plot for variant rs12959877 on chromosome 18. Abbreviations: ADNI, Alzheimer's Disease Neuroimaging Initiative; BIOCARD, Predictors of Cognitive Decline Among Normal Individuals; BLSA, Baltimore Longitudinal Study of Aging; chr, chromosome; GWAS, genome‐wide association study; MAP, Memory and Aging Project; MTG, middle temporal gyrus transcallosal tract; NACC, National Alzheimer's Coordinating Center; VMAP, Vanderbilt Memory and Aging Project; ROS, Religious Orders Study; WRAP, Wisconsin Registry for Alzheimer's Prevention.

A cross‐sectional GWAS for each dMRI metric of each tract was performed using linear regression models covarying for sex, age, and the first three ancestral principal components, followed by a meta‐analysis across cohorts (Table S6 for meta‐analysis summary statistics [*p* < 0.05]). Figure 2 presents an ideogram illustrating the 500 most significant genomic signals based on the smallest *p*‐value associated with WM microstructure. In total, six genome‐wide significant loci for different tract‐by‐microstructure combinations were discovered. We identified a locus with 38 genome‐wide significant (*p* < 5 × 10^−8^) SNPs (Figure 3, lead SNP: rs12959877, effect allele frequency [EAF] = 0.44, β = 0.002 ± 3.18 × 10^−4^, *p* = 5.78 × 10^−9^, intronic *CDH19*) for MTG RD_FWcorr_, with additional suggestive associations (*p* < 1 × 10^−5^) at this site for RD_FWcorr_ of STG and ITG. When evaluating eQTL (GTEx Portal) evidence, we found that all 38 SNPs were eQTLs for the gene *CDH19* in lung and spleen tissue (Table S7). This gene is highly expressed in oligodendrocytes and functions as a calcium‐dependent cell adhesion glycoprotein.^59^ Additional genome‐wide significant signals were found in proximity to the genes *KC6*, *SENP5*, *RORA*, *FAM107B*, and *MIR548A1* (Table 2).

**TABLE 2:** Statistics for genome‐wide significant SNPs (*p* < 5×10^−8^).

Tract	dMRI	SNP	Nearest gene	EAF	Beta	SE	*p*‐value
MTG	RD_FWcorr_	rs12959877; for additional 37 SNPs see Table S4a	*CDH19*	0.44	0.002	3.18 × 10^−4^	5.78 × 10^−9^
ITG	FA_FWcorr_	rs79952731	*KC6*	0.02	−0.013	0.002	7.37 × 10^−9^
ITG	FA_FWcorr_	rs142917632	*KC6*	0.02	−0.013	0.002	7.37 × 10^−9^
MTG	FA_FWcorr_	rs79952731	*KC6*	0.02	−0.012	0.002	2.58 × 10^−9^
MTG	FA_FWcorr_	rs142917632	*KC6*	0.02	−0.012	0.002	2.58 × 10^−9^
STG	FA_FWcorr_	rs79952731	*KC6*	0.02	−0.015	0.002	6.53 × 10^−11^
STG	FA_FWcorr_	rs142917632	*KC6*	0.02	−0.015	0.002	6.53 × 10^−11^
STG	AxD_FWcorr_	rs79952731	*KC6*	0.02	−0.020	0.004	1.95 × 10^−8^
STG	AxD_FWcorr_	rs142917632	*KC6*	0.02	−0.020	0.004	1.95 × 10^−8^
ILF	FA_FWcorr_	rs8026709	*RORA*	0.36	0.003	0.001	8.94 × 10^−9^
ILF	AxD_FWcorr_	rs8026709	*RORA*	0.36	0.004	0.001	5.31 × 10^−9^
STG	RD_FWcorr_	rs11542181	*FAM107B*	0.03	0.005	0.001	2.64 × 10^−8^
Cingulum	RD_FWcorr_	rs56017587	*MIR548A1*	0.13	0.002	4.25 × 10^−4^	2.98 × 10^−8^
Fornix	AxD_FWcorr_	rs78407651	*SENP5*	0.03	−0.012	0.002	4.77 × 10^−8^

Abbreviations: AxD, axial diffusivity; dMRI, diffusion MRI; EAF, effect allele frequency; FA, fractional anisotropy; FW, free water; ILF, inferior longitudinal fasciculus; ITG, inferior temporal gyrus transcallosal tract; MD, mean diffusivity; MTG, middle temporal gyrus transcallosal tract; RD, radial diffusivity; SE, standard error; SNP, single nucleotide polymorphism; STG, superior temporal gyrus transcallosal tract.

To contextualize the identified loci, we queried the Oxford Brain Imaging Genetics Server (BIG40)^51^ and ENIGMA‐VIS.^52^ At a nominal threshold (*p* < 0.05), our genome‐wide variants showed associations with diverse imaging‐derived phenotypes, including dMRI measures, resting‐state functional MRI measures, cortical thickness, and regional and tissue volume. However, none of the variants attained suggestive‐level or genome‐wide significance in these resources, indicating that our locus‐WM trait associations likely represent novel findings pending replication (Table S8).

### 3.3 Significant associations between gene expression of RORA, FAM107B, and KC6 with cognitive function and AD pathology

To evaluate the clinical and neuropathologic relevance of the genes identified in the GWAS analysis, we investigated the associations of *CDH19*, *KC6*, *SENP5*, *RORA*, *FAM107B*, and *MIR548A1* expression profiles in the PCC, DLPFC, and CN with cognitive measures (cross‐sectional and longitudinal cognition) and AD pathologies (tau tangles, neuritic plaques, Aβ, neurofibrillary tangles).

**FIGURE 4:**
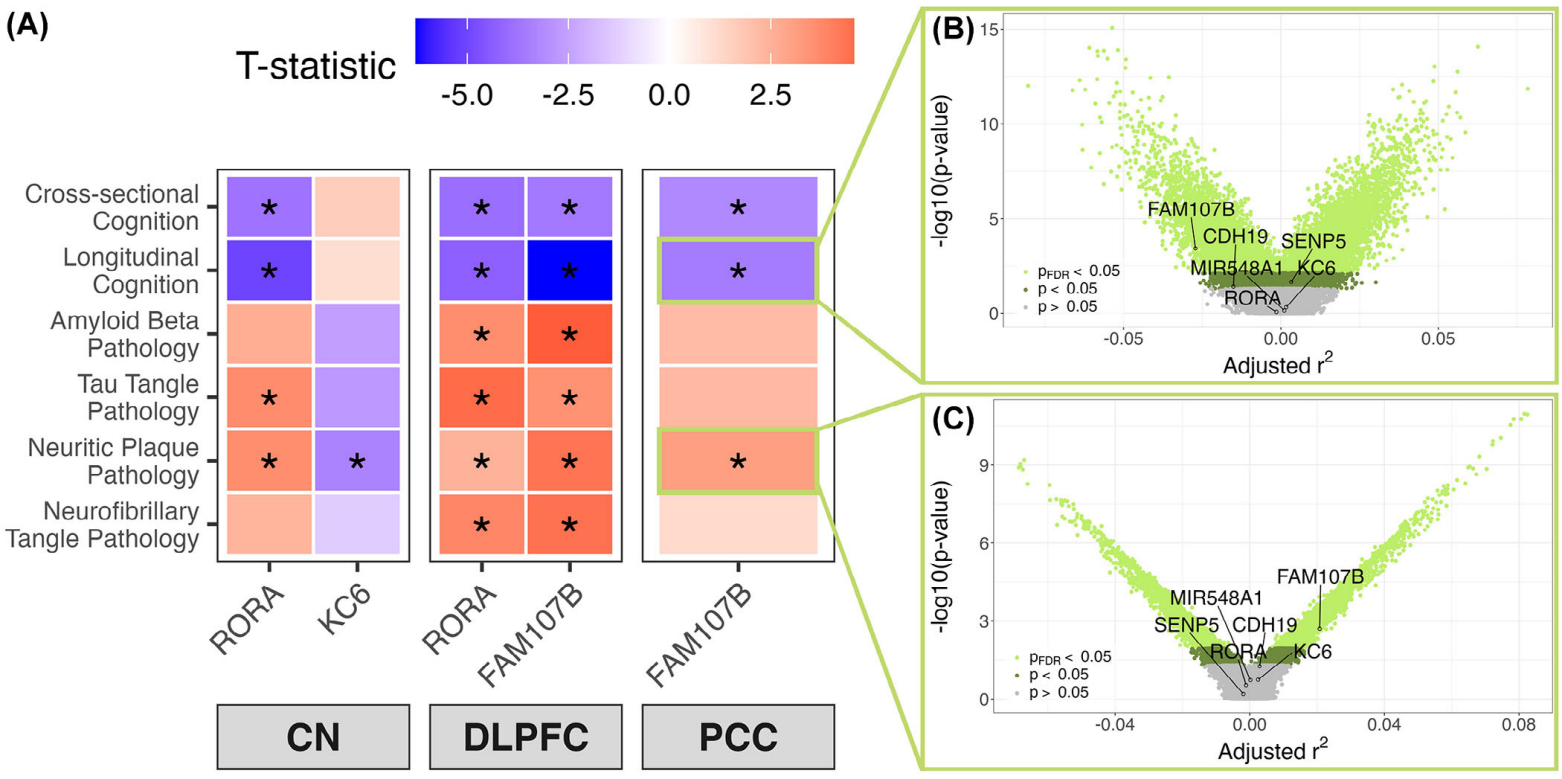
Gene expression profiles in brain tissues of genes identified through GWAS. (A) Heatmap displaying t‐statistics for associations between gene expression profiles in three brain tissues for GWAS‐identified genes (*RORA, SENP5, KCNK6, CDH19, FAM107B*, and *MIR548A1*) with cognitive outcomes and AD pathologies. Only genes with at least one significant association (*p_FDR_* < 0.05) with a cognitive or AD outcome are included. (B,C) Exemplary volcano plots showing the association of *FAM107B* expression in PCC with (B) longitudinal cognitive performance and (C) neuritic plaque burden. Significance thresholds are indicated by color. All volcano plots can be found in Figure S1. Abbreviations: AD, Alzheimer's disease; CN, caudate nucleus; DLPFC, dorsolateral prefrontal cortex; FDR, false discovery rate; GWAS, genome‐wide association study; PCC, posterior cingulate cortex.

Significant associations were observed for *RORA*, *FAM107B*, and *KC6* (Figure 4, Figure S1). Associations for *RORA* expression included cross‐sectional cognition in CN (β = −0.654 ± 0.173; *p_FDR_* = 0.011) and DLPFC (β = −0.570 ± 0.145; *p_FDR_* = 0.001), and longitudinal cognition in CN (β = −0.082 ± 0.016; *p_FDR_* = 4.39 × 10^−5^) and DLPFC (β = −0.570 ± 0.014; *p_FDR_* = 1.31 × 10^−3^). Significant AD pathologies for *RORA* included tau tangles in CN (β = 0.751 ± 0.204; *p_FDR_* = 0.010) and DLPFC (β = 0.756 ± 0.165; *p_FDR_* = 7.95 × 10^−5^), neurite plaques in CN (β = 0.294 ± 0.081; *p_FDR_* = 0.010) and DLPFC (β = 0.165 ± 0.066; *p_FDR_* = 0.049), neurofibrillary tangles in DLPFC (β = 0.194 ± 0.051; *p_FDR_* = 0.002), and Aβ in DLPFC (β = 0.515 ± 0.143; *p_FDR_* = 0.003).

For *FAM107B* gene expression, there were significant associations with cross‐sectional cognition in DLPFC (β = −0.242 ± 0.066; *p_FDR_* = 0.003) and PCC (β = −0.259 ± 0.081; *p_FDR_* = 0.015), longitudinal cognition in DLPFC (β = −0.038 ± 0.006; *p_FDR_* = 2.21 × 10^−8^) and PCC (β = −0.025 ± 0.007; *p_FDR_* = 0.005). AD pathologies included tau tangles in DLPFC (β = 0.263 ± 0.075; *p_FDR_* = 0.003), neuritic plaques in DLPFC (β = 0.130 ± 0.030; *p_FDR_* = 3.43 × 10^−4^) and PCC (β = 0.119 ± 0.038; *p_FDR_* = 0.018), neurofibrillary tangles in DLPFC (β = 0.102 ± 0.023; *p_FDR_* = 2.55 × 10^−4^), and Aβ in DLPFC (β = 0.325 ± 0.065; *p_FDR_* = 3.98 × 10^−5^). Furthermore, *KC6* expression in CN was associated with neurite plaques (β = −0.055 ± 0.017; *p_FDR_* = 0.019). All results for this analysis can be found in Table S9.

### 3.4 Gene‐ and pathway‐level analyses highlight biological mechanisms related to immune function, neurotrophic signaling, and cardiovascular traits

The genetic architecture of WM microstructure was also investigated at the gene and pathway level. Gene‐level analyses revealed a significant association between cingulum AxD_FWcorr_ and the gene *SERPINA12* (*z* = 4.60, *p_FDR_* = 0.038, N_SNPs_ = 132), which is known to influence obesity and atherosclerosis by modulating glycolipid metabolism.^60^, ^61^, ^62^ Table S10 depicts the statistics for all gene analyses (*p* < 0.05).

Pathway analysis identified significant pathways (*p_FDR_* < 0.05) for ITG FA_FWcorr_, STG FA_FWcorr_ and UF AxD_FWcorr_. Enriched pathways for ITG FA_FWcorr_ were related to immune function, encompassing isotype switching of B cells, B cell–mediated immunity, and the production of immunoglobulins and antibodies. Beyond immune‐related pathways, STG FA_FWcorr_ was associated with several neurotrophic signaling pathways involving neurotrophic receptor tyrosine kinease 3 (NTRK3) and NTRK2 via the RAS pathway, which are crucial for nervous system development and survival. This tract was also linked to insulin‐like growth factor 1 receptor (IGF1R) signaling and insulin receptor binding pathways. Furthermore, STG FA_FWcorr_ showed associations with cancer mechanisms, including mutant forms and internalization of the epidermal growth factor receptor (EGFR). It also included pathways related to the overexpression of the human epidermal growth factor receptor 2 (HER2) and signaling events mediated by the EphA2 receptor. For UF AxD_FWcorr_, enriched pathways included photoreceptor cell outer segment organization and the binding of the peptide hormone angiotensin to its receptor. Statistics for all pathway analyses can be found in Table S11 (*p* < 0.05).

### 3.5 Genetic covariance between WM microstructure with cardiovascular, lipid, inflammatory, and neurological traits

**FIGURE 5:**
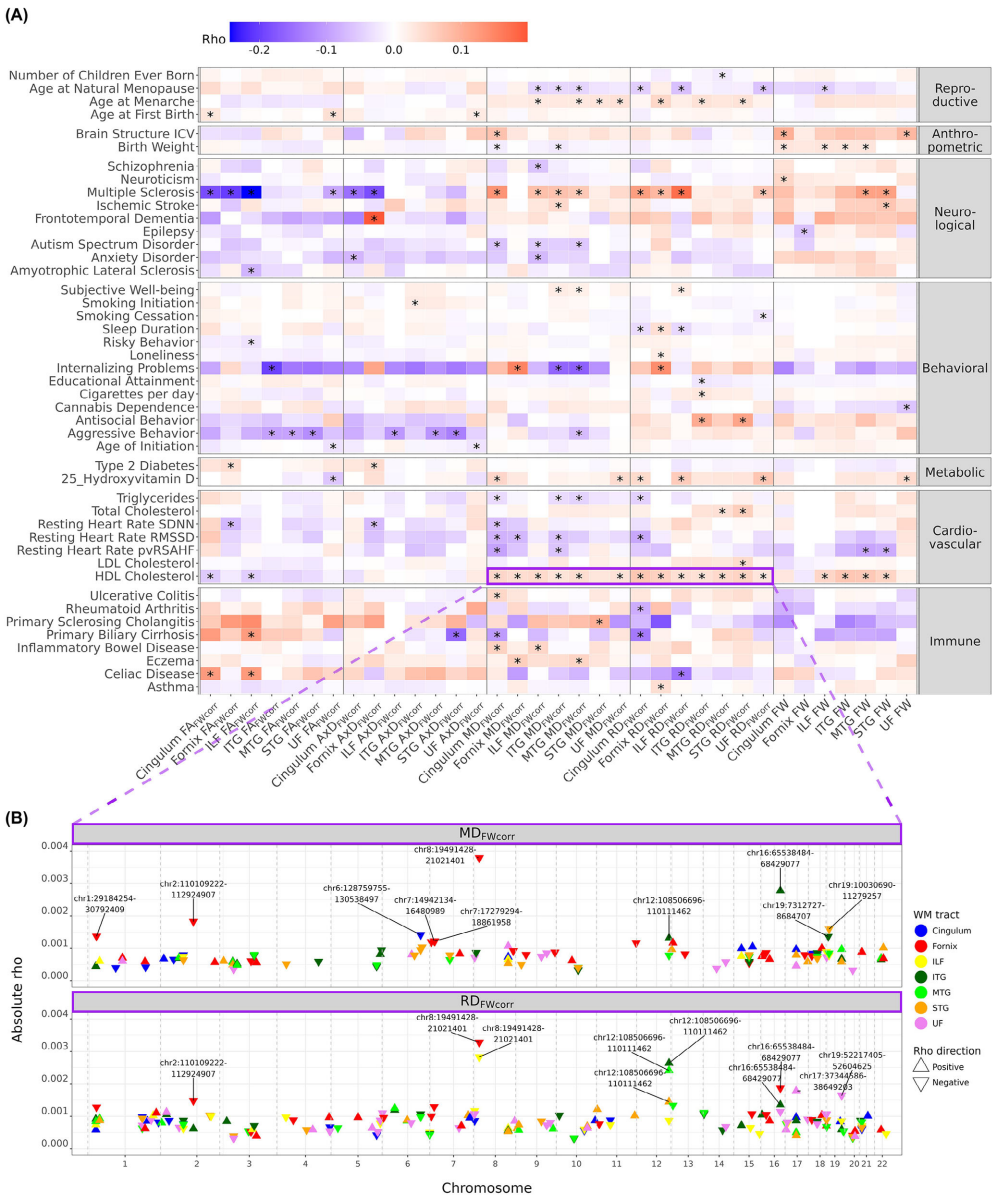
Genetic covariance between complex traits and white matter (WM) microstructure. (A) Genetic covariance between diffusion MRI (dMRI) metrics and complex traits that have shown at least one association with a dMRI metric (*p_FDR_* < 0.05). “*” marks a *p_FDR_* < 0.05. (B) Local genetic covariance between HDL cholesterol with MD_FWcorr_ and RD_FWcorr_. Only genomic regions with a *p* < 0.05 were included. Abbreviations: AxD, axial diffusivity; FA, fractional anisotropy; FDR, false discovery rate; FW, free water; ILF, inferior longitudinal fasciculus; ITG, inferior temporal gyrus transcallosal tract; MD, mean diffusivity; MTG, middle temporal gyrus transcallosal tract; RD, radial diffusivity; STG, superior temporal gyrus transcallosal tract; UF, uncinate fasciculus.

To investigate the extent of shared genetic factors between WM microstructure and complex traits (*N* = 65), we performed genetic covariance analyses using GNOVA. Traits that exhibited at least one FDR‐corrected significant association with one dMRI metric are displayed in Figure 5A. HDL cholesterol demonstrated multiple significant genetic associations with WM microstructure. Specifically, we observed negative covariance with FA_FWcorr_ and AxD_FWcorr_, alongside positive covariance with MD_FWcorr_, RD_FWcorr_, and FW. When examining the local genetic covariance for HDL cholesterol with MD_FWcorr_ and RD_FWcorr_, multiple distributed genomic regions exhibiting significant covariation were identified (Figure 5B, Table S12).

The genetics of resting heart rate variability traits (SDNN, RMSSD, pvRSAHF) and triglyceride showed negative associations with those of MD_FWcorr_, RD_FWcorr_, and FW. For metabolic traits, type 2 diabetes was positively associated with fornix FA_FWcorr_ and AxD_FWcorr_, whereas hydroxyvitamin D demonstrated several positive associations with MD_FWcorr_ and RD_FWcorr_. Moreover, multiple genetic associations between WM microstructure were identified with immune‐related diseases, including ulcerative colitis, rheumatoid arthritis, primary sclerosing cholangitis, primary biliary cirrhosis, inflammatory bowel disease, eczema, celiac disease, and asthma. Neurological and psychiatric traits also showed significant associations, encompassing schizophrenia, neuroticism, ischemic stroke, frontotemporal dementia, epilepsy, autism spectrum disorder, anxiety disorder, and amyotrophic lateral sclerosis. Behavioral traits such as subjective well‐being, smoking initiation, smoking cessation, cigarettes per day, cannabis dependence, sleep duration, risky behavior, loneliness, internalizing problems, educational attainment, antisocial behavior, aggressive behavior, and age of initiation furthermore shared significant genetic architecture with WM microstructural traits. Statistics for all computed tests can be found in Table S13. Most of the genetic covariance results remained significant and showed similar effect directions after removing the *APOE* region from the genome (Figure S2, Table S14).

Genetic covariance analysis between FW‐corrected dMRI metrics and 3143 neuroimaging‐derived phenotypes from the UK Biobank identified 9218 significant associations after FDR correction of 110,005 tests performed (Table S15). Figure S3 highlights the neuroimaging‐derived phenotypes with at least 10 significant genetic associations across the FW‐corrected dMRI metrics. Notably, substantial genetic overlap was observed with the UK Biobank dMRI measures (e.g., mode of the arcuate fasciculus, FA_CONV_ of the posterior corona radiata) and resting‐state network metrics (e.g., NET100_0452, NET100_1425).

### 3.6 TWAS identified an association between STG RDFWcorr and DNAJB14

The summary statistics derived from the meta‐analyzed GWAS of WM microstructure were used to compute TWAS using S‐PrediXcan. A significant association between STG RD_FWcorr_ and the gene *DNAJB14* in colon transverse tissue (*z* = −5.47, *p_FDR_* = 0.016, N_SNPs_ = 27) was identified. *DNAJB14* is involved in chaperone cofactor‐dependent protein refolding and protein‐containing complex assembly.^63^ Results (*p* < 0.05) for all analyses can be found in Table S16.

## 4 DISCUSSION

This study investigated the genetic architecture of limbic WM microstructure in a large, harmonized sample of older adults enriched for cognitive impairment, utilizing advanced FW‐corrected dMRI metrics. We observed substantial heritability of limbic WM microstructure, especially within tracts such as the cingulum, fornix, and ILF. Our meta‐analyzed GWAS identified several genetic loci associated with WM characteristics, implicating the genes *CDH19*, *KC6*, *SENP5*, *RORA*, *FAM107B*, and *MIR548A1*. Furthermore, we identified gene‐level association of WM microstructure with *DNAJB14* using TWAS and with *SERPINA12* using gene‐level analysis. Pathway and genetic covariance analyses revealed significant links between limbic WM microstructure and biological processes related to inflammation, vascular health, lipid metabolism, and various neurological conditions.

The strongest GWAS signal identified was a locus on chromosome 18, with most SNPs acting as eQTLs for *CDH19*. This gene, also known as cadherin 19, encodes a calcium‐dependent cell‐adhesion glycoprotein highly expressed in oligodendrocytes and Schwann cells, which are crucial for myelin formation and maintenance.^59^ This finding suggests a novel link between genetic variation influencing myelin‐related cell adhesion processes and WM microstructural integrity in older adults. Another association was found near *SENP5* on chromosome 3 with fornix AxD_FWcorr_. *SENP5* regulates post‐translational SUMOylation, critical for gene expression, DNA repair, and mitochondrial dynamics, and has been implicated previously in synaptogenesis and synaptic function in mature neurons.^64^, ^65^ Based on our findings, *SENP5* seems to be also involved in WM microstructural differences in later life.

Several other GWAS‐identified genes, including *RORA, FAM107B*, and *KC6*, showed significant associations of their brain tissue expression levels with cognitive decline and neuropathologies (tau tangles, neurite plaques, neurofibrillary tangles, and Aβ plaques) in our bulk RNA‐seq analyses. *RORA* encodes a protein that binds to hormone response elements upstream of multiple genes to enhance their expression. It regulates key genes involved in neurological functions and is implicated in autism spectrum disorders, highlighting its role in neuronal differentiation and synaptic plasticity.^66^ As a nuclear receptor transcription factor, *RORA* interacts with transcriptional regulators like insulin and brain‐derived neurotrophic factors, both implicated in neurodegeneration and cognitive aging, including AD.^67^ In addition, *RORA* supports neuronal survival and has a neuroprotective role in Parkinson's disease by protecting neurons from oxidative stress.^68^ *FAM107B* is widely expressed in the brain and associated with various brain structural features, such as cortical thickness, subcortical volume, and overall brain morphology.^69^ *KC6* is a less well‐characterized RNA gene and has been associated with corneal biology and diseases.^70^ Although canonical AD‐risk genes did not reach genome‐wide significance, prior work in the same cohorts using a hypothesis‐driven approach demonstrated associations between limbic FW‐corrected WM microstructure and established AD‐risk variants, including *APOE*, *TMEM106B*, *PTK2B*, and *WNT3*, as well as AD polygenic risk.^27^ Overall, these findings indicate that some of the identified genes are involved in general brain health, which is further supported by the observed genetic associations between WM microstructure and neurological and psychiatric conditions, such as multiple sclerosis, aggressive behavior, and internalizing problems.

Our study strongly implicates inflammatory, lipid metabolism, and vascular mechanisms in limbic WM health. *RORA* is also involved in regulating cholesterol and glucose metabolism and blood vessel morphogenesis.^67^ *SENP5*, in addition to its importance for brain health, has a critical role in cardiac function by regulating mitochondrial balance. Excessive de‐SUMOylation by *SENP5*, which is upregulated in human heart failure, has been linked to cardiomyopathies and cardiac dysfunction.^71^, ^72^ Our post‐GWAS analyses supported associations between WM microstructure with lipid and vascular mechanisms, revealing gene‐level associations for *SERPINA12* and links to insulin and inflammation pathways. Notably, *SERPINA12* is elevated in individuals with obesity and is associated with obesity‐linked metabolic traits, such as insulin resistance, impaired glycemic control, and cardiovascular disease.^73^ The connection between WM and cardiovascular mechanisms was also demonstrated by genetic overlap between WM microstructure and HDL cholesterol, triglycerides, resting heart rate variability traits, ischemic stroke, type 2 diabetes, and several inflammatory diseases. These findings suggest that cardiovascular risk and associated systemic inflammation may provide a pathophysiological basis for WM alterations observed in later life, which can in turn contribute to risk for neurodegenerative diseases and cognitive decline. Vascular conditions such as hypertension, atherosclerosis, and type 2 diabetes may compromise vascular integrity, leading to reduced blood flow or microvascular damage in the brain. Such vascular challenges adversely affect WM, potentially resulting in axonal and myelin damage. This highlights the importance of intact energy metabolism and the cardiovascular system in brain health. Although FW‐corrected metrics provide improved tissue specificity relative to conventional diffusion measures, they remain sensitive to broader biological processes affecting WM. Consistent with our genetic covariance analyses, which demonstrated overlap with vascular, lipid, inflammatory, and neurological traits, the identified loci likely reflect pathways influencing WM integrity through both microstructural and systemic mechanisms, including neurodegenerative and cerebrovascular processes. Further research is needed to disentangle signals related to vascular mechanisms from AD‐specific pathologies.

A key strength of this study is the investigation of genetic influence on WM microstructure within well‐characterized cohorts of older individuals, including those with cognitive impairment, which enhances our ability to uncover biological mechanisms relevant to aging and neurodegenerative diseases. Unlike large‐scale cohorts and studies such as UK Biobank and ENIGMA that have predominantly focused on WM in midlife, our study specifically addresses the genetic architecture of WM degeneration in the context of aging, cognitive impairment, and AD risk. In addition, the use of FW‐corrected dMRI metrics provides a more accurate quantification of WM microstructure by mitigating confounds from extracellular FW.

This study has several limitations. Our analyses were conducted on non‐Hispanic White individuals, which, while reducing confounding from population stratification, limits the generalizability of our findings to more diverse populations. Future research should aim to replicate these findings in larger, multi‐ethnic cohorts. The cross‐sectional and correlational nature of our analyses precludes causal inferences. Although the use of single‐shell dMRI data allowed for the inclusion of a large, harmonized dataset, the FW correction approach used here is based on a bi‐tensor model whose parameter estimation is generally better constrained in multi‐shell than in single‐shell diffusion acquisitions.^74^ Accordingly, although FW‐corrected metrics improve tissue specificity relative to conventional diffusion measures, indices derived from single‐shell data remain model dependent and should be interpreted as nonspecific tissue‐compartment diffusion measures rather than direct markers of a single biological process. Future studies employing multi‐shell dMRI approaches, such as Neurite Orientation Dispersion and Density Imaging (NODDI), could provide more detailed insights into distinct tissue compartments and potentially quantify aspects like neuroinflammation.^74^ Although FW correction reduces partial volume effects from extracellular FW, structural MRI measures such as regional brain volumes and WM hyperintensity (WMH) burden were not incorporated in the present study. As both macrostructural atrophy and WMH burden can influence diffusion‐derived metrics, we cannot exclude residual confounding, and the identified associations may reflect a combination of microstructural, neurodegenerative, and cerebrovascular processes. Finally, only a small subset of our cohort (5%) had a formal clinical diagnosis of AD at the time of imaging. Enriching future cohorts with individuals across the AD spectrum may enhance the detection of neurodegeneration‐specific genetic signals.

Taken together, this imaging genetics study identified several novel genes linked to limbic WM microstructure in multiple, harmonized aging cohorts. Notably, bulk RNA‐seq analyses demonstrated that some of these genes were associated with cognitive decline and Aβ and tau burden, suggesting AD relevance. Furthermore, our findings revealed that the genetic architecture of limbic WM is strongly tied to vascular health and inflammation, highlighting these pathways as promising avenues for future therapeutic development.

## CONFLICT OF INTEREST STATEMENT

S.C.J. has received consulting fees from Enigma Biomedical and has served on an advisory board for ALZPath within the past 2 years. A.J.S. has received support from Avid Radiopharmaceuticals, a subsidiary of Eli Lilly, and has participated in scientific advisory boards for Bayer Oncology, Eisai, Novo Nordisk, and Siemens Medical Solutions. A.J.S. has also served on the Observational Study Monitoring Board for the Multi‐Ethnic Study of Atherosclerosis (MESA) Medical Imaging with Deep Learning, an NIH NHLBI‐funded study. E.C.M. has received consulting fees from Eli Lilly. B.B.B. has received consulting fees from New Amsterdam, Cognito Therapeutics, and Merry Life Biomedical. B.B.B. has also served on advisory boards for the Weston Advisory Board, Rush Alzheimer's Disease Research Center External Advisory Board, Emory Alzheimer's Disease Research Center External Advisory Board, and South Texas Alzheimer's Disease Research Center External Advisory Board. B.B.B. is the founder of Cognovance, LLC. T.J.H. has received consulting fees from Circular Genomics and has served on the scientific advisory board of Vivid Genomics. A.L.J. has served as Chair of the Observational Study Monitoring Board for the Diverse‐VCID, and has also served as an advisory board member for Lantheus Diagnostic and Therapeutic Innovations. B.L. has received consulting fees from Silver Maple LLC, serves as a board member for Medical Imaging with Deep Learning (MIDL). S.R. has served on the external advisory board of the Adult Aging Brain Connectome and on independent scientific advisory boards for the Canadian Consortium on Neurodegeneration in Aging and Dementias Platform UK. A.W.T. serves on the RHU‐SHIVA and ADNI steering committees. All other authors declare no competing interests. Author disclosures are available in the Supporting Information.

## CONSENT STATEMENT

All participants provided written informed consent in their respective cohort studies.

## Supporting information



Supporting Information

Supporting Information

Supporting Information

## Footnotes

* Data used in preparation of this article were obtained from the Alzheimer's Disease Neuroimaging Initiative (ADNI) database (adni.loni.usc.edu). As such, the investigators within the ADNI contributed to the design and implementation of ADNI and/or provided data but did not participate in analysis or writing of this report. A complete listing of ADNI investigators can be found at: http://adni.loni.usc.edu/wp-content/uploads/how_to_apply/ADNI_Acknowledgement_List.pdf
