# Supplementary material for: Genetic architecture of the limbic white matter microstructure in aging and Alzheimer's Disease
Source: Alzheimers Dement. 2026 Jul 6;22(7):e71630. doi: 10.1002/alz.71630 (PMC13338108; doi:10.1002/alz.71630)
Supplement: Supplementary file 2 — Supporting Information [file ALZ-22-e71630-s003.docx]

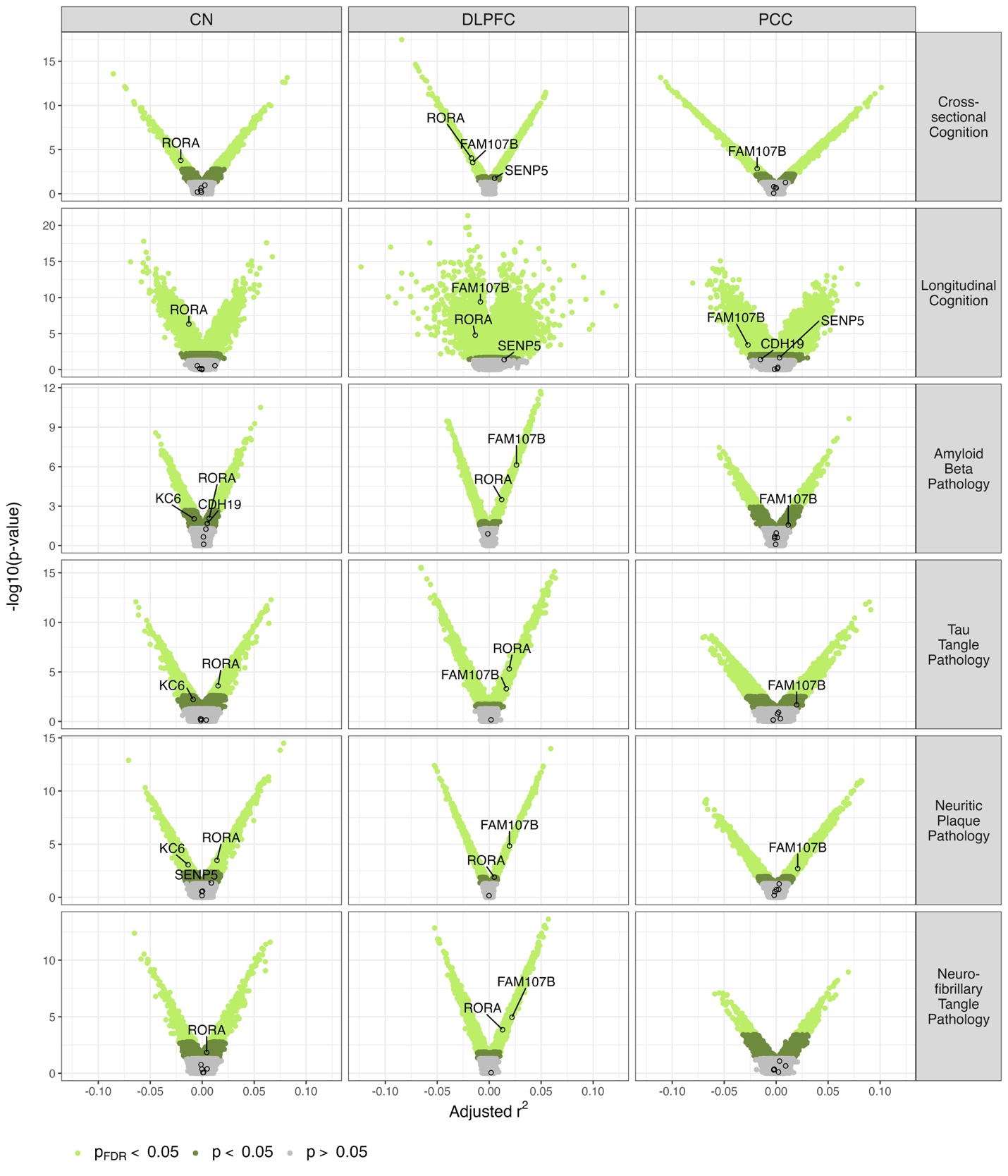
**SUPPLEMENTAL FIGURE S1.** **Gene expression profiles in brain tissues of genes identified through GWAS.**

Volcano plots of gene expression profiles in brain tissues for the genes *RORA*, *SENP5*, *KC6*, *CDH19*, *FAM107B*, and *MIR548A1* identified through GWAS associated with cognitive outcomes and AD pathologies. Significance thresholds are indicated by color. Highlighted genes with a *p* < 0.05 are labeled. Abbreviations: AD, Alzheimer’s Disease; CN, caudate nucleus; DLPFC, dorsolateral prefrontal cortex; GWAS, genome-wide association study; FDR, false discovery rate; PCC, posterior cingulate cortex.

**
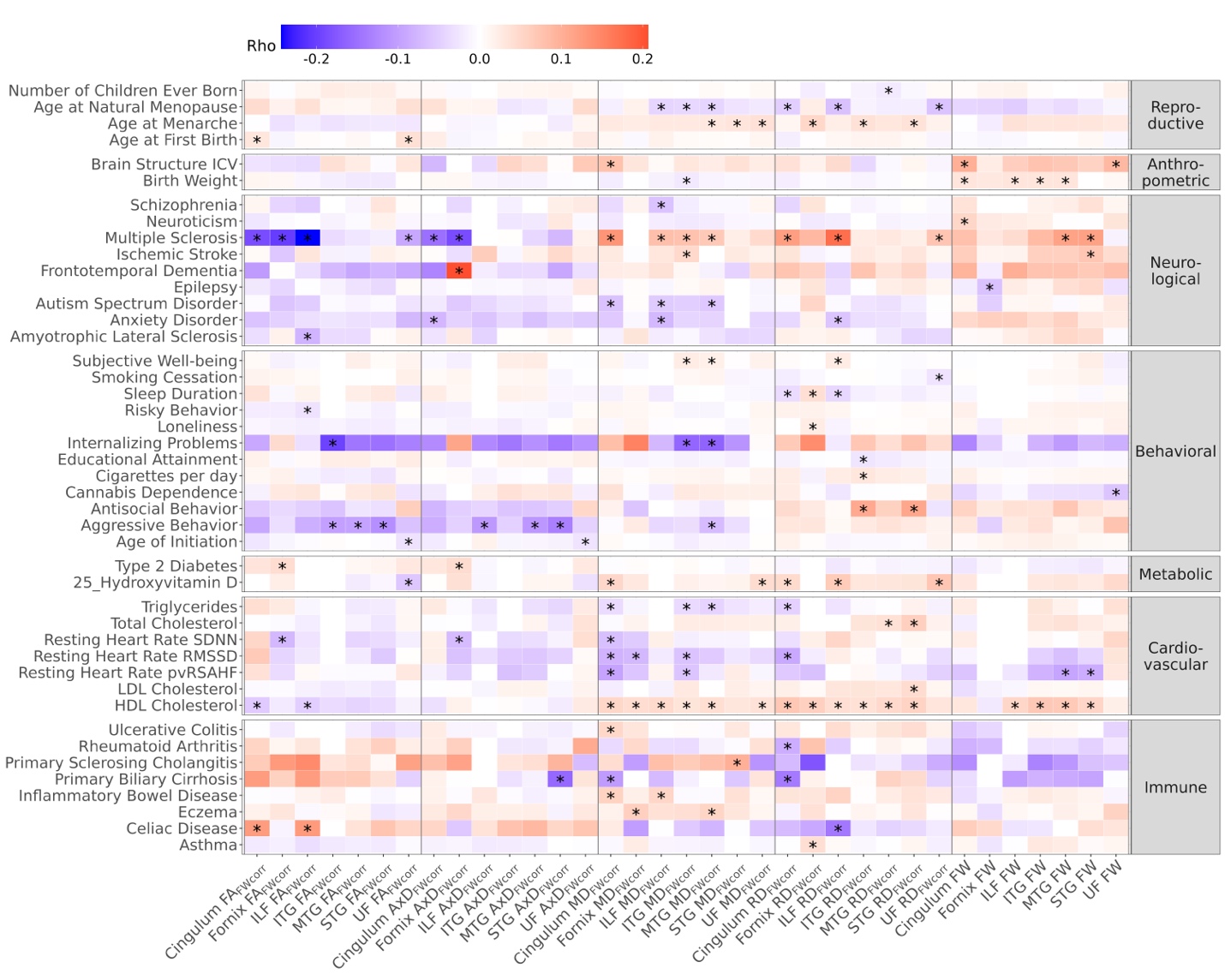
SUPPLEMENTAL FIGURE S2. Genetic covariance between complex traits and WM microstructure when the *APOE* region is removed from the genome.**

Genetic covariance between dMRI metrics (x-axis) and complex traits and diseases (y-axis) when the *APOE* region is removed from the genome. The traits included have shown at least one FDR-significant association with a dMRI metric. "*" marks genetic covariance with a *p_FDR_* < 0.05. Abbreviations: AxD, axial diffusivity; FA, fractional anisotropy; FDR, false discovery rate; FW, free water; ILF, inferior longitudinal fasciculus; ITG, inferior temporal gyrus transcallosal tract; MD, mean diffusivity; MTG, middle temporal gyrus transcallosal tract; RD, radial diffusivity; STG, superior temporal gyrus transcallosal tract; UF, uncinate fasciculus.


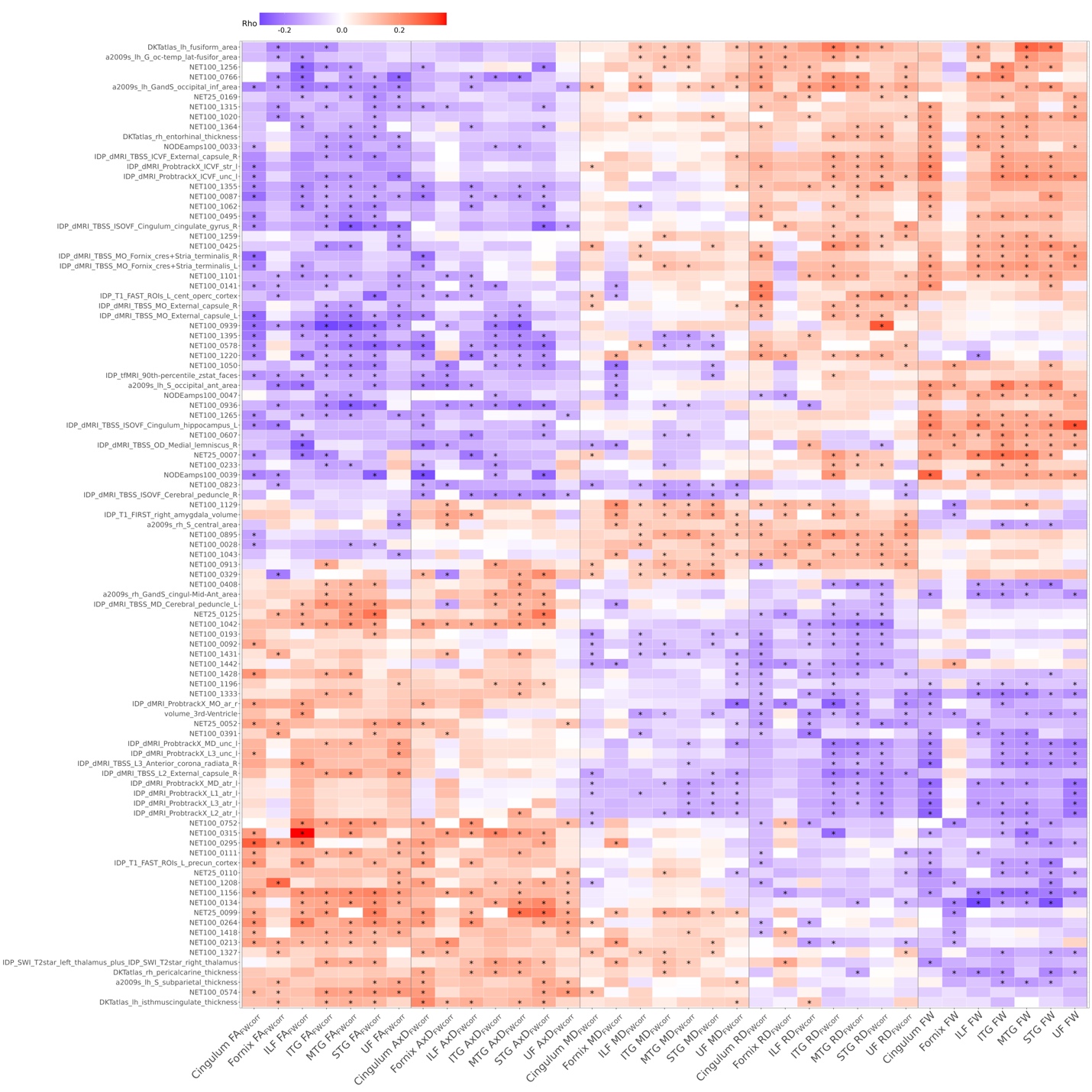
**SUPPLEMENTAL FIGURE S3. Genetic covariance between brain traits and WM microstructure.**

Genetic covariance between dMRI metrics (x-axis) and brain traits (y-axis). The traits included have shown at least ten FDR-significant associations with a dMRI metric. "*" marks genetic covariance with a *p_FDR_* < 0.05. The y-axis is clustered using Euclidean distance. Abbreviations: AxD, axial diffusivity; FA, fractional anisotropy; FDR, false discovery rate; FW, free water; ILF, inferior longitudinal fasciculus; ITG, inferior temporal gyrus transcallosal tract; MD, mean diffusivity; MTG, middle temporal gyrus transcallosal tract; RD, radial diffusivity; STG, superior temporal gyrus transcallosal tract; UF, uncinate fasciculus.
